# Facing the conundrum: which first-line therapy should be used for patients with metastatic triple-negative breast cancer carrying germline *BRCA* mutation?

**DOI:** 10.37349/etat.2023.00198

**Published:** 2023-12-27

**Authors:** Sabah Alaklabi, Arya Mariam Roy, Lubna N. Chaudhary, Shipra Gandhi

**Affiliations:** Institute of Experimental Endocrinology and Oncology “G. Salvatore”-National Research Council (IEOS-CNR), Italy; Nicola Normanno, Istituto Nazionale Tumori-IRCCS-Fondazione G. Pascale, Italy; ^1^Department of Medical Oncology, Cancer Center of Excellence, King Faisal Specialist Hospital and Research Center, Riyadh 11211, Saudi Arabia; ^2^Department of Medicine, Roswell Park Comprehensive Cancer Center, Buffalo, NY 14203, USA; ^3^Division of Hematology/Oncology, Froedtert and Medical College of Wisconsin, Milwaukee, WI 53226, USA

**Keywords:** Metastatic triple-negative breast cancer, programmed cell death ligand-1, Poly (ADP-ribose) polymerase inhibitor, sequencing of therapy, targeted therapy

## Abstract

Pembrolizumab combined with chemotherapy has been established as the preferred first-line therapy for treating metastatic triple-negative breast cancer (mTNBC) with programmed cell death ligand-1 (PD-L1)-positive disease since its approval for that indication. However, the optimal sequencing of therapy remains an unanswered question for a subset of mTNBC patients who harbor germline breast cancer gene 1/2 (*BRCA1/2*; *gBRCA1/2*) mutation. This article aims to offer insights into the optimal therapy sequencing for mTNBC patients with *gBRCA1/2* mutations and its impact on clinical decision-making. The perspective offered is based on the best currently available data and propose a practical algorithm to guide the management of this subgroup in the frontline setting.

## Introduction

Breast cancer (BC) mutations are strongly linked to triple-negative BC (TNBC). Around 20% of people with TNBC have a germline mutation in the BC gene (*BRCA*) [1]. When compared to non-carriers, *BRCA1* carriers had considerably lower 10-year overall survival (OS) [2]. This underscores the need for development of specific management guidelines tailored to this population. The optimal sequencing strategy for first-line treatment in a subset of metastatic TNBC (mTNBC) patients with both programmed cell death ligand-1 (PD-L1)-positive disease and germline *BRCA1/2* (*gBRCA1/2*) mutation remains a topic of ongoing debate. A recent post hoc exploratory analysis of the OlympiAD study has suggested potential survival benefit of olaparib when administered in the first line [3]. The durability of response of immune checkpoint inhibitors (ICIs) and their limited efficacy if used in later lines of therapy have led to the preference for ICIs in the frontline setting. However, the selection between ICIs and Poly (ADP-ribose) polymerase (PARP) inhibitors as the initial therapy for this subgroup of patients remains a complex clinical decision.

In this article, the objective is to provide guidance to practicing oncologists on the most appropriate treatment sequence for this specific patient population by proposing a sequencing strategy for ICIs and PARP inhibitors in mTNBC and *gBRCA1/2* mutations that takes into account available evidence and clinical perspectives.

## PD-L1 as a target in TNBC

PD-L1 expression is more prevalent in TNBC compared to other subtypes of BC, mainly on inflammatory immune cells and occasionally on neoplastic cells [4]. Higher percentage of tumor or immune cells expressing PD-L1 predicts response to ICIs [5–7]. Multiple trials investigated the role of ICIs in the treatment of metastatic BC (MBC) with *BRCA* mutation. KEYNOTE 355 showed a 7-month median OS extension in favor of pembrolizumab-chemotherapy in previously untreated mTNBC [hazard ratio (HR) 0.73; 95% confidence interval (CI) 0.55–0.95, *P* = 0.0093] [7]. Given the OS benefit, ICIs have become the new standard of care in patients with mTNBC and combined positive score (CPS) ≥ 10.

IMpassion130, a phase III randomized trial, investigated atezolizumab and nab-paclitaxel in mTNBC [8, 9]. The co-primary endpoints included progression-free survival (PFS) and OS in the intention to treat (ITT) population. The study design followed a hierarchy that allowed for the assessment of OS in the PD-L1-positive population only if a significant improvement in OS was observed in the ITT population. In the ITT population, the median OS was 21.0 months (95% CI 19.0–23.4 months) with atezolizumab and nab-paclitaxel, and 18.7 months (95% CI 16.9–20.8 months) with placebo and nab-paclitaxel (HR 0.87; 95% CI 0.75–1.02; *P* = 0.077). Exploratory analysis in the PD-L1-positive subgroup had a median OS of 25.4 months (95% CI 19.6–30.7 months) in the atezolizumab and nab-paclitaxel arm and 17.9 months (95%, 13.6–20.3 months) in the placebo arm (HR 0.67; 95% CI 0.53–0.86). Based on the IMpassion130 trial, accelerated approval to atezolizumab in combination with chemotherapy was granted by the Food and Drug Administration (FDA) in March 2019. The primary endpoint of PFS superiority in PD-L1 positive mTNBC patients was not met in IMpassion131 (HR 0.82; 95% CI 0.60–1.12; *P* = 0.20). Additionally, no OS benefit was observed in either PD-L1-positive or ITT patients [10]. As a result of IMpassion131 disappointing results, Roche withdrew atezolizumab’s USA mTNBC indication [11].

In the context of clinical trials pertaining to immunotherapy efficacy in mTNBC, KEYNOTE 119 investigated single-agent pembrolizumab *versus* investigator’s choice of chemotherapy in the second- or third-line setting in mTNBC. The trial showed no significant improvement in OS with pembrolizumab in later settings [12, 13]. KEYNOTE 086 examined pembrolizumab monotherapy in mTNBC patients. It had two cohorts: cohort A (second line or later) and cohort B (frontline setting). In cohort A, the majority of patients had disease progression with an objective response rate (ORR) of 5.3%, while cohort B had a 21.4% ORR to single agent pembrolizumab in the frontline setting. Both trials reported greater clinical benefit with higher PD-L1 expression, which is consistent with prior knowledge [14, 15].

The phase II part of KEYNOTE 150 results, which specifically looked at the combination of eribulin and pembrolizumab for patients with mTNBC, were in line with the previous observations. It enrolled both chemotherapy naive patients for their metastatic disease and patients who had undergone one or two lines of chemotherapy. Again, the findings called into question whether or not ICIs are truly of benefit outside the first line setting in mTNBC [16].

Although we have seen exciting results with ICIs in mTNBC, the current approval for ICIs is only limited to PD-L1 positive group. Data coming from new clinical trials may result in expansion of ICIs indication irrespective of PD-L1 positivity. For example, the recent update from the phase Ib/II basket study BEGONIA, the combination of the trophoblast cell surface antigen 2 (TROP-2) antibody-drug conjugate datopotamab deruxtecan (Dato-DXd) with the ICI durvalumab (anti-PD-L1); and also, trastuzumab durextecan with durvalumab showed promising efficacy with manageable adverse events in the first line setting in mTNBC [17, 18].

## PARP as a target in TNBC

Germline mutations in *BRCA1* and *BRCA2* are present in 9–18% of TNBCs [19]. The *BRCA1* and *BRCA2* pathways function to promote DNA double-strand break repair by homologous recombination, maintaining genomic stability, and inhibiting carcinogenesis [20–23]. When the *BRCA*-associated DNA repair pathway HR becomes defective, alternative DNA repair mechanisms dependent on PARP enzymes, take over [24, 25]. When combined with the loss of DNA repair via *BRCA*-dependent mechanisms, inhibiting PARP leads to synthetic lethality and cell death [26, 27].

## Clinical data of efficacy of PARP inhibition in *BRCA* mutated TNBC

The FDA approved talazoparib and olaparib in 2018 to treat advanced human epidermal growth factor receptor 2 (HER2)-negative BC with a *gBRCA1/2* mutation [28, 29]. Talazoparib approval was based on EMBRACA, that randomized BC patients with germline mutation in *BRCA*, and locally advanced or MBC to talazoparib or chemotherapy. The primary endpoint of PFS was met with a median of 8.6 months in the talazoparib arm compared to 5.6 months in the chemotherapy arm (HR 0.54; 95% CI 0.41–0.71; *P* < 0.0001). Talazoparib had no OS improvement over chemotherapy. The reported median OS was 19.3 months in the talazoparib arm *versus* 19.5 months in the chemotherapy arm (HR 0.848; 95% CI 0.670–1.073; *P* = 0.17) [30, 31].

**Figure 1:**
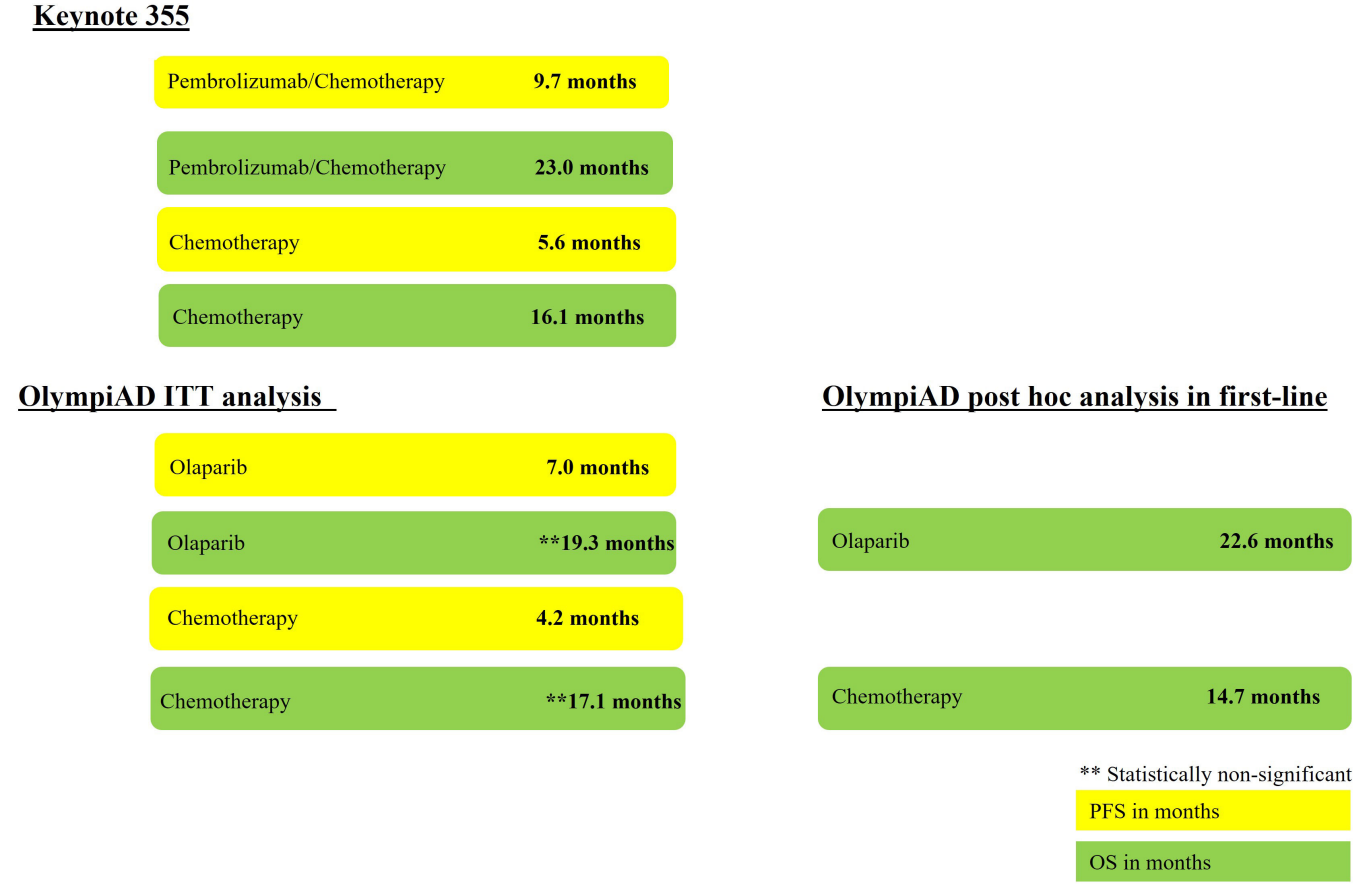
Summary of PFS and OS benefit from Keynote 355 and OlympiAD

Olaparib was studied in MBC with a *gBRCA1/2* mutation [3, 32, 33]. The FDA approval was granted based on data from OlympiAD, an open-label clinical trial that randomized 302 patients with *gBRCA* mutation, HER2-negative MBC (50% were TNBC), to olaparib or chemotherapy. PFS was the primary endpoint which was met with a reported median of 7.0 months in the olaparib arm, compared with 4.2 months in the chemotherapy arm (HR 0.58; 95% CI 0.43–0.80; *P* = 0.0009) [32]. OS was a secondary endpoint in OlympiAD and there was no statistically significant difference in median OS between olaparib and chemotherapy in the overall population (HR 0.90; 95% CI 0.63–1.29; *P* = 0.57). Recently, a post hoc analysis suggested that olaparib may have long term survival benefit when administered frontline with reported OS benefit of nearly 8 months (22.6 months *versus* 14.7 months; HR 0.55; 95% CI 0.33–0.95) [3]. Even though this was an unplanned analysis, it highlights the importance of conducting studies to guide the optimal sequence of therapies in TNBC with *BRCA* mutation. The reported PFS and OS benefits from KEYNOTE 355 and OlympiAD are summarized in Figure 1.

## Combination of platinum and PARP inhibitors in *BRCA*-mutated MBC

The efficacy of platinum and PARP inhibitors combination for treatment of mTNBC with *BRCA* mutation has been investigated in light of the evidence showing correlation between *BRCA1/2* gene mutations and increased sensitivity to platinum-based chemotherapy [34, 35]. The first large randomized phase III study to investigate platinum salts in TNBC was Triple-Negative Breast Cancer Trial (TNT). In this study, mTNBC patients received either carboplatin or docetaxel as first-line treatment. No significant difference in ORR was observed in the ITT population, however, subgroup analysis based on *BRCA* mutational status revealed that *BRCA*-mutated patients treated with carboplatin had a significantly higher ORR and longer PFS compared to those treated with docetaxel [36]. Another study, which included both cisplatin and carboplatin as treatment options in the 1st and 2nd line, showed that *BRCA*-mutated TNBC patients had a higher ORR compared to those without *BRCA* mutations [37].

Combining platinum and PARP inhibitors in *BRCA* mutant MBC has been investigated. BROCADE3 evaluated the efficacy of veliparib *versus* placebo when combined with carboplatin and paclitaxel, with monotherapy continuation upon discontinuation of carboplatin and paclitaxel, in HER2-negative advanced BC patients with a *gBRCA1/2* mutation. Patients were randomized assigned to veliparib plus carboplatin-paclitaxel or placebo plus carboplatin-paclitaxel. Adding veliparib improved the median PFS to 14.5 months (95% CI 12.5–17.7), compared to 12.6 months (95% CI 10.6–14.4) in the control group (HR 0.71; *P* = 0.0016). The findings support the combination of platinum-based chemotherapy and veliparib in patients with *gBRCA* mutation, given the improvement in PFS [38]. In terms of toxicity, 34% of patients experienced serious adverse events in the veliparib group, compared to 29% in the placebo group.

## In patients with mTNBC and *gBRCA* mutation, which frontline treatment should be used?

With the current approval of ICIs in the neoadjuvant/adjuvant setting for stage II–III TNBC (KEYNOTE 522), and PARP inhibitors in the adjuvant setting for TNBC patients with residual disease and *gBRCA* mutation (OLYMPIA trial), deciding about optimal treatment in the metastatic setting with the same drugs is challenging due to lack of data [39, 40]. Olaparib could be considered upfront for TNBC patients with *gBRCA1/2* mutations who had a complete pathological response to neoadjuvant pembrolizumab yet relapsed within a year of adjuvant pembrolizumab completion.

**Figure 2:**
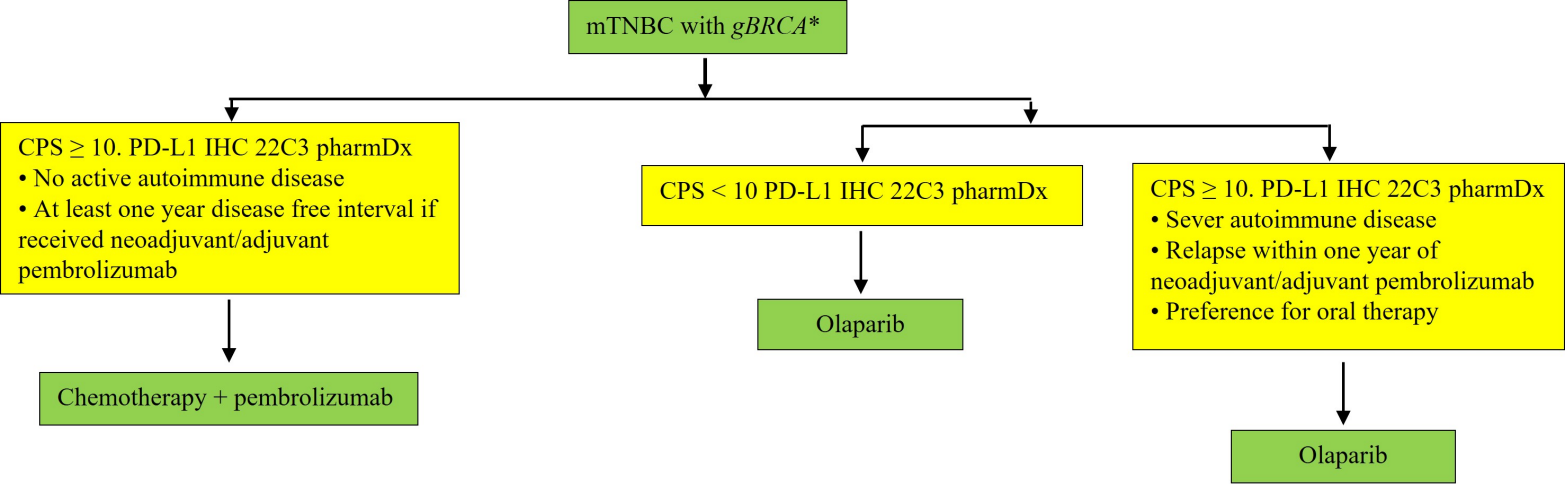
Practical guidance on management of mTNBC patients who are gBRCA mutation carriers. ^*^ This algorithm could also be used for the management of mTNBC with sBRCA and gPALB2 mutations based on findings from the TBCRC 048 clinical trial showing improved outcomes with use of olaparib in these subgroups. gPALB2: germline partner and localizer of BRCA2; sBRCA: somatic BRCA

If a patient is a *gBRCA1/2* mutation carrier with PD-L1 CPS ≥ 10 mTNBC, we still prefer to use ICIs first given the consistency of data showing durability of response that is likely to be achieved in the frontline setting with an intact immune system, and confirmed OS benefit [7, 13–15, 41]. There is also a real risk of losing immunotherapy benefit if it is postponed to later lines, whereas PARP inhibitors have PFS benefit even in later-line settings. Although the OlympiAD extended follow-up data indicate an OS benefit in the frontline setting, this analysis was post hoc, which has inherent limitations and lacks statistical power, thus results must be interpreted with caution. Based on the above, it is crucial to obtain information on PD-L1 status for appropriate management in first-line setting with PD-L1 inhibitors. The proposed algorithm in Figure 2 provides a framework for therapy in mTNBC patients who are *gBRCA* mutation carriers. Of note, a recent Italian BC expert consensus on the management of mTNBC aligns with our viewpoint [42].

Finally, treatment decisions should be tailored to each patient’s unique risk of adverse events, preferences, and available treatment resources.

In conclusion, this paper highlights the challenges in selecting frontline treatment for mTNBC patients with *gBRCA* mutation and emphasizes the need for future trials to directly compare ICIs and PARP inhibitors in this specific patient population. Such trials would provide essential insights to guide clinical decision-making and optimize treatment outcomes.

While Table 1 summarizes practical differences between ICIs and olaparib, we caution the reader that this is not a cross-trial comparison, and the current paper is not intended to conduct any comparative analysis between trials.

**Table 1:** Comparison of pembrolizumab/chemotherapy *versus* olaparib from a practical standpoint

**Comparative aspect**	**Olaparib**	**Pembrolizumab/Chemotherapy**
Clinical trial	OlympiAD	Keynote 355
Eligibility in mTNBC	*gBRCA*, *sBRCA*, gPALB2^*^	CPS ≥ 10
Response rate reported	60%	41%
Median duration of response	6.4 Months (IQR, 2.8 to 9.7) in the olaparib group compared to 7.1 months (IQR, 3.2 to 12.2) in the standard chemotherapy group	12.8 Months (95% CI 9.9–25.9) in pembrolizumab/chemotherapy group compared to 7.3 months (95% CI 5.5–15.4) in the chemotherapy group
Median time to onset of response	47 Days	Not reported
Median PFS	7.0 Months *versus* 4.2 months in the olaparib group compared to the standard-therapy group respectively (HR 0.58; 95% Cl 0.43–0.80; *P* < 0.001).	9.7 Months with pembrolizumab-chemotherapy compared to 5.6 months with placebo-chemotherapy respectively; HR 0.65, 95% CI 0.49–0.86; one-sided *P* = 0.0012 (co-primary objective met)
OS	No OS benefit in the ITT. OS of 22.6 months compared to 14.7 months if used in the 1st line setting (HR 0.55; 95% CI 0.33–0.95, *P* = 0.02)^**^	OS of 23.0 months compared to 16.1 months in the placebo-chemotherapy group (HR 0.73; 95% CI 0.55–0.95; *P* = 0.0093)
Contraindications	MDS/AML Pneumonitis	Active severe autoimmune disease
Possible adverse effects	Myelosuppression, nausea, vomiting, risk of MDS/AML, pneumonitis	Any autoimmune adverse events including but not limited to irreversible hypophysitis, adrenal insufficiency, thyroiditis, fatal cardiomyositis
Convenience	Oral therapy requiring monthly follow up to monitor	IV therapy every 3 weeks

^*^ Patients with HER2 negative MBC with gPALB2 or sBRCA1/2 mutations were found to benefit from treatment with olaparib according to TBCRC 048, a phase II trial of olaparib for MBC and homologous recombination-related gene mutations; ^**^ post hoc analysis. IQR: interquartile range; MDS: myelodysplastic syndrome; AML: acute myeloid leukemia; IV: intravenous

## Abbreviations

BC: breast cancer
BRCA: breast cancer gene
CI: confidence interval
CPS: combined positive score
FDA: Food and Drug Administration
gBRCA1/2: germline breast cancer gene 1/2
HER2: human epidermal growth factor receptor 2
HR: hazard ratio
ICIs: immune checkpoint inhibitors
ITT: intention to treat
MBC: metastatic breast cancer
mTNBC: metastatic triple-negative breast cancer
ORR: objective response rate
OS: overall survival
PARP: Poly (ADP-ribose) polymerase
PD-L1: programmed cell death ligand-1
PFS: progression-free survival
sBRCA: somatic breast cancer gene
TNBC: triple-negative breast cancer

## Declarations

### Copyright

© The Author(s) 2023.
